# Multivariate Normal Distribution Method for a Virtual Cerebral Arterial Population

**DOI:** 10.1002/cnm.70117

**Published:** 2025-11-15

**Authors:** Kazuyoshi Jin, Ko Kitamura, Shunji Mugikura, Naoko Mori, Stephen Payne, Makoto Ohta, Hitomi Anzai

**Affiliations:** ^1^ Institute of Fluid Science, Tohoku University Sendai Japan; ^2^ Graduate School of Biomedical Engineering Tohoku University Sendai Miyagi Japan; ^3^ Division of Image Statistics, Tohoku University Tohoku Medical Megabank Organization Sendai Japan; ^4^ Department of Radiology Akita University Graduate School of Medicine Akita Japan; ^5^ Institute of Applied Mechanics, National Taiwan University Taipei City Taiwan ROC

**Keywords:** cerebrovascular, data augmentation, multivariate normal distribution, synthetic population, virtual population

## Abstract

Recently, the concept of a virtual population (Vpop) has attracted attention to provide large‐scale, diverse datasets without compromising individual privacy. The development of the Vpop modelling method for the cerebrovasculature shape is necessary to be established with simple parameter tuning and post‐processing. This study introduces a multivariate normal distribution (MVND) method to generate a Vpop for the cerebrovasculature shape. We defined an MVND by using the position and inner radius, which represent the vascular shape (centerline), as variables. Patient‐specific arteries (basilar artery and internal carotid artery) obtained from MR images were used as a real population (Rpop) to generate an MVND. Then, virtual arteries were sampled from this MVND to generate a Vpop. To evaluate the validity of this method for reproducing shape diversity, we calculated the geometrical features of the centerline in each population. The centerline shows qualitatively similar characteristics between Vpop and Rpop. Geometrical features such as average length calculated from Vpop are in the same range as those of Rpop. Moreover, the distribution of geometrical features exhibits a good degree of fit between Vpop and Rpop. Since MVND considers the correlation among all position and inner radius variables, centerline continuity and anatomical characteristics of cerebrovasculature can be automatically included. Hence, geometric features and their distribution can be reproduced without any parameter tuning. The consistency in geometric parameters between the two populations supports the validity of the MVND method and indicates the potential for generating a Vpop for the cerebrovasculature in a more straightforward and simplified manner.

## Graphical abstract

A simple multivariate normal distribution (MVND) method for generating a population of distributed virtual arteries with multiple geometric features was developed. The distribution of geometric features in virtual arteries represents the distribution in real arteries.

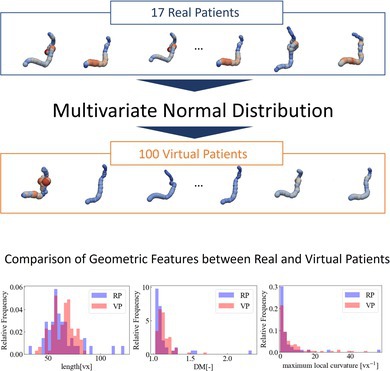

## 1 Introduction

Cerebrovascular diseases, including stroke and aneurysms, remain a significant global health concern, with their diagnosis and treatment often relying heavily on accurate imaging and analysis of the affected brain vasculature [1]. The highly complex and varied nature of cerebrovascular shapes poses a considerable challenge in developing robust diagnostic tools and treatment strategies [2, 3, 4, 5]. To address this challenge, there is a need for large, diverse datasets of brain vascular structural networks.

However, obtaining such extensive datasets from real patients is often impractical due to privacy concerns, ethical considerations, and the high costs associated with data collection and annotation [6, 7, 8]. Moreover, real‐world datasets are inherently limited in size and cannot guarantee coverage of the diverse anatomical variations found in the human body. As a result, real‐world data may not capture the full spectrum of possible vascular shape variations.

As a way to overcome these limitations, data augmentation [9] techniques are often used in this domain. In this context, medical data augmentation denotes the fusion of genuine, real‐world patient (RP) data and artificially generated, virtual patient (VP) data, aiming to boost the efficacy of AI models in categorization tasks. In medical imaging, techniques such as mirroring and cropping data, which aim to amplify the volume of training data using virtual data, have been involved to refine the efficacy of prevailing deep learning models for semantic segmentation. In studies for 3D shapes of blood vessels, the statistical shape model using principal component analysis (PCA‐SSM) has proved to be one of the most common for generating VP. For instance, Thamsen et al. utilized SSM to generate vascular models to study the influence of vessel shape on hemodynamic [10]. In addition, the point distribution method (PDM) and Gaussian process morphable models (GPMMs) have been used in studies of virtual reproduction of face and hand shape [11]. GPMMs offer a more flexible and generalizable approach to shape modelling compared to traditional PCA‐SSM. In GPMMs, it is common to set the landmark points, to calculate the covariance, and to determine the number of principal components in the same way as PCA‐SSM. It is also necessary to determine the regular parameter in the fitting algorithm. However, to the best of our knowledge, GPMM has not yet been applied to vascular modelling.

Expanding the concept from the generation of individual data to the generation of population synthesis, the concept of virtual populations (Vpop) has been developed as a new means of data augmentation technology. Vpop allows the creation of statistically representative datasets that mimic actual populations. Because Vpop can provide large‐scale, diverse datasets without compromising individual privacy, research fields such as machine learning, which rely on such datasets, are paying significant attention to Vpop [12, 13]. In earlier studies, the statistical Vpop modelling method has been applied not in shape but in the joint distribution of clinical data and physiological measurements (such as age, gender, smoking status, blood pressure, etc.) in pharmacometrics. Techniques such as bootstrapping have been employed as statistical Vpop modelling methods. Each VP can be obtained by sampling multiple points from a Vpop that is represented as a continuous distribution. However, extracting realizable VP from the probabilistic statistical methods necessitates complex parameter tuning, including the implementation of intricate exclusion criteria. Consequently, the methods generating a large volume of VP for training purposes, such as in deep learning applications, present considerable challenges.

To tackle these problems, a statistical Vpop generation method with simplified parameter tuning is available to facilitate large‐scale VP generation. For example, the multivariate normal distribution (MVND) and its counterpart, the multivariate log‐normal distribution can be effective as one of the simplest statistical modelling methods to generate VP with few parameter tunings. These were first introduced by Tannenbaum et al. for generating biological covariates to reproduce the characteristics of real patient populations [14]. For instance, Teutonico et al. applied this MVND method for Vpop based on actual clinical data including age, gender, and smoking status without any parameter tunings [15].

The aim of the present study is to generate a large amount of virtual artery shape data. To achieve this, parameter tuning and post‐processing (e.g., inclusion) should be simple when extracting (generating) virtual arteries from the mathematical model. The present study based on the MVND concept also calculates the covariance matrix but uses the entire covariance matrix for mathematical modelling without dimensionality reduction. Hence the MVND may contain more dimensionality to catch the subtle variations among patients compared to classical PCA‐SSM or GPMMs. The MVND in the present study was randomly sampled directly from a multivariate distribution (*x, y, z, r*), which is one of the phenotypes for shape. With this simple method, we can generate for the first time a Vpop of vessel centerline coordinates and radius data, which are referred to as VPs in the present study. These VPs would be expected to have a similar distribution to that of the RPs, through quantitative evaluation of the agreement between RPs and VPs. In addition, the reproduction of real and virtual BA and ICA shapes was verified through visualization. Particularly for the ICA, which has a more complex course compared to the BA, the distinctive anatomical shapes such as siphon shape were visualized to qualitatively check their plausibility. The real and virtual geometric features of BA centerlines and ICA centerlines, the main regions of the cerebral vasculature, were calculated and then the histograms of the geometric features were compared between RPs and VPs.

In Section 2, the dataset, generation method, the evaluation procedures, and the methods used to calculate geometric features are described. In Section 3, the results of this simulation are described. In Section 3.1, the visualizations of generated virtual artery and real artery are shown to intuitively assess whether the appearance reproduced the features specific to the actual vessel shape. In Sections 3.2 and 3.3, length and inner diameter results of real and virtual artery are compared with those reported in previous studies to evaluate how plausible our virtual arteries are as individual shapes. In Section 3.4, the distributions of geometric features for virtual artery and real artery are compared by histograms to evaluate how plausible our virtual arteries are as groups. In Section 3.5, the Kullback–Leibler divergence distributions for each landmark point are shown to further examine how many landmark points are necessary to reproduce realistic artery shape. In Section 4, the discussions of the simulations are presented. In Section 5, the conclusion is summarized.

## 2 Methods

### 2.1 Data Acquisition of Original Real Population

The study was approved by the Tohoku University Ethics Committee (2020‐1‐478). The database comprised 46 hypertensive patients aged 31–76 years (56.29 ± 10.63 years, 27 men and 19 women) with primary aldosteronism (PA).

The centerline data obtained from magnetic resonance angiography (MRA) images [16] were used. The centerlines were extracted by thresholding after registering the MRAs of 46 patients to brain atlas, MNI152 [17]. After extraction, centerlines were annotated on a bifurcation basis. The ICA is typically divided into 7 segments based on its anatomical course and relationships with surrounding structures. These segments are often referred to using the Bouthillier classification [18]. The cervical segment (C1), petrous segment (C2), lacerum segment (C3), cavernous segment (C4), clinoid segment (C5), ophthalmic segment (C6) and communicating segment (C7) in the real ICA are also represented in the virtual ICA. In this study, the C1 segment, located extracranially, is not visualized well in MRA; therefore, the analysis focuses on segments C2 through C7. The data included 17 left ICA (L_ICA), 16 right ICA (R_ICA), and 46 BA, with details of the numbers of centerline points shown in Table 1. The artery radius was calculated at each datapoint based on the inscribed sphere [16]. Details of the process can be found in the Supporting Information.

**TABLE 1:** The number of centerline data points in each part.

	BA	L_ICA	R_ICA
Average	53.4	123.6	127.4
S.D.	12.9	12.9	12.5
Max	100	151	158
Min	31	101	96
Median	51	124	126

### 2.2 Generation Method

**FIGURE 1:**
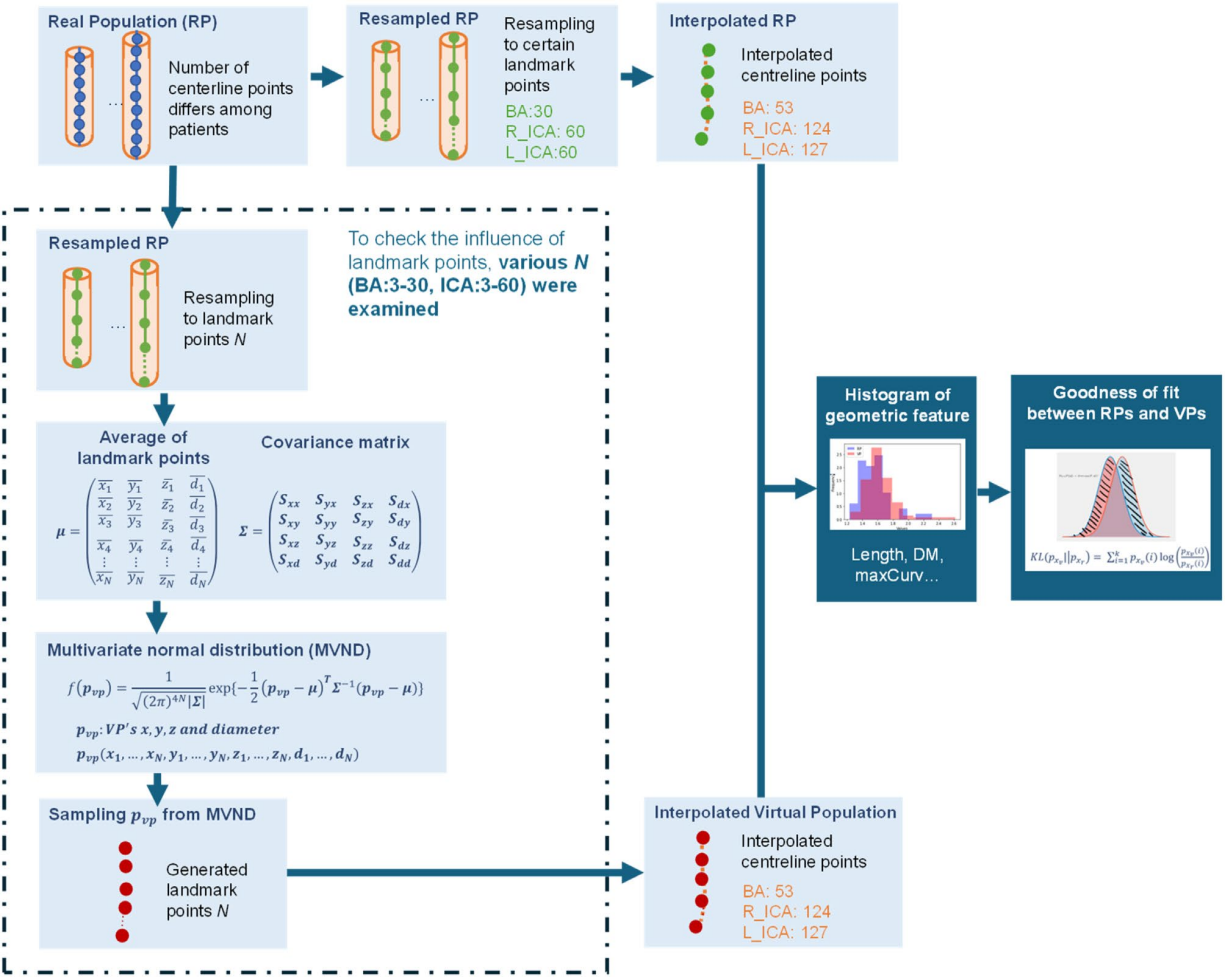
The schematics of VP generation and evaluating process.

Figure 1 represents the schematics of the VP generation and evaluating process. The MVND method was adopted for the generation of VPs because MVND is the simplest statistical method for describing a distribution. We defined MVNDs separately on L_ICA, R_ICA, and BA for each. MVND is defined as an extension of the one‐dimensional normal distribution and is calculated from the mean matrix and covariance matrix. To apply this method, the centerlines were represented by (*x, y, z, r*) values as variables. The centerline was equally sampled into k landmark points, including both ends. Hence, the artery for one patient can be represented by a one‐dimensional vector as follows,

(1) $$p=\left(x_{1},\ldots ,x_{k},y_{1},\ldots ,y_{k},z_{1},\ldots ,z_{k},r_{1},\ldots ,r_{k}\right).$$

A set of arteries was expressed using a sample size of patients *N* as:

(2) $$\begin{array}{c} P=\left\{p_{1}^{T},\cdots ,p_{N}^{T}\right\}. \end{array}$$

From ***P***, the mean matrix ***μ*** and variance–covariance matrix ***Σ*** were calculated as (3) and (4), respectively.

(3) $$\begin{array}{c} \mu =\frac{1}{N}\sum_{i=1}^{N}p_{i} \end{array}.$$

(4) $$\begin{array}{c} \Sigma =\left(\begin{array}{ccc} \sigma_{x_{1},x_{1}} & \cdots & \sigma_{x_{1},r_{k}}\\ \vdots & \ddots & \vdots \\ \sigma_{d_{k},x_{1}} & \cdots & \sigma_{d_{k},r_{k}} \end{array}\right) \end{array}.$$

In this context, $\sigma_{m,n}$ represents the covariance between variable m and variable n (where m and n refer to the elements of $p_{i}$). Note that $\bar{m}$ and $\bar{n}$, as defined above, differ in terms of whether they represent the element‐wise mean of μ within ***p***, or the overall mean matrix of the ***p*** matrix.

(5) $$\begin{array}{c} \sigma_{m,n}=\frac{1}{N-1}\sum_{i=1}^{N}\left(m_{i}-\bar{m}\right)\left(n_{i}-\bar{n}\right). \end{array}$$

Finally, the MVND of ***p*** (4k‐elements) can be expressed as:

(6) $$\begin{array}{c} f\left(p\right)=\frac{1}{{\left(2\pi \right)}^{2k}{\left|\Sigma \right|}^{\frac{1}{2}}}\exp\left\{-\left(p-\mu \right)\Sigma^{-1}\left(p-\mu \right)\right\}, \end{array}$$

where ***Σ*** ^**−1**^ is the Moore–Penrose pseudoinverse of ***Σ***. ***Σ*** ^***−1***^ is estimated using singular value decomposition (SVD). To verify this MVND assumption by such as Mardia's test, the sample size of patients (N) must be higher than the number of ***p***‐elements (4k).

Under the definition of the MVND as shown in (6), we generated a set of virtual arteries based on a set of virtual landmark points by random sampling of the MVND, thus (*x*, *y*, *z*, *r*) values in the landmark points follow the MVND (see in Figure 1). The numbers of landmark points were defined from the numbers of average centerline points in Table 1. These numbers of average centerline points, 124 landmark points for L_ICA, 127 landmark points for R_ICA, and 53 landmark points for BA, were set to match the numbers of average data points of RPs. Subsequently, quadratic B‐spline interpolation [19] was performed on these points to suppress noises and reconstruct a more natural representation of the vessel centerlines since the number of landmark points alone was too few to represent the vessel centerlines. The curve after B‐spline interpolation was then defined as the VP.

### 2.3 Evaluation Method

At each number of landmark points, 30 sets of 100 VPs were generated to simulate the necessary diversity among the RPs for L_ICA, R_ICA, and BA such that 3000 VPs were generated. Diversity was evaluated using the geometric features described in the next section. Reproducibility was verified through these 30 sets, with each set containing 100 VPs.

Furthermore, to facilitate a comparison between the VPs and the RPs, because the number of RP's centerline points is different, quadratic B‐spline interpolation was also applied to the RPs. The number of interpolation points on RPs was set to be the same as for the VPs.

#### 2.3.1 Geometric Features

**FIGURE 2:**
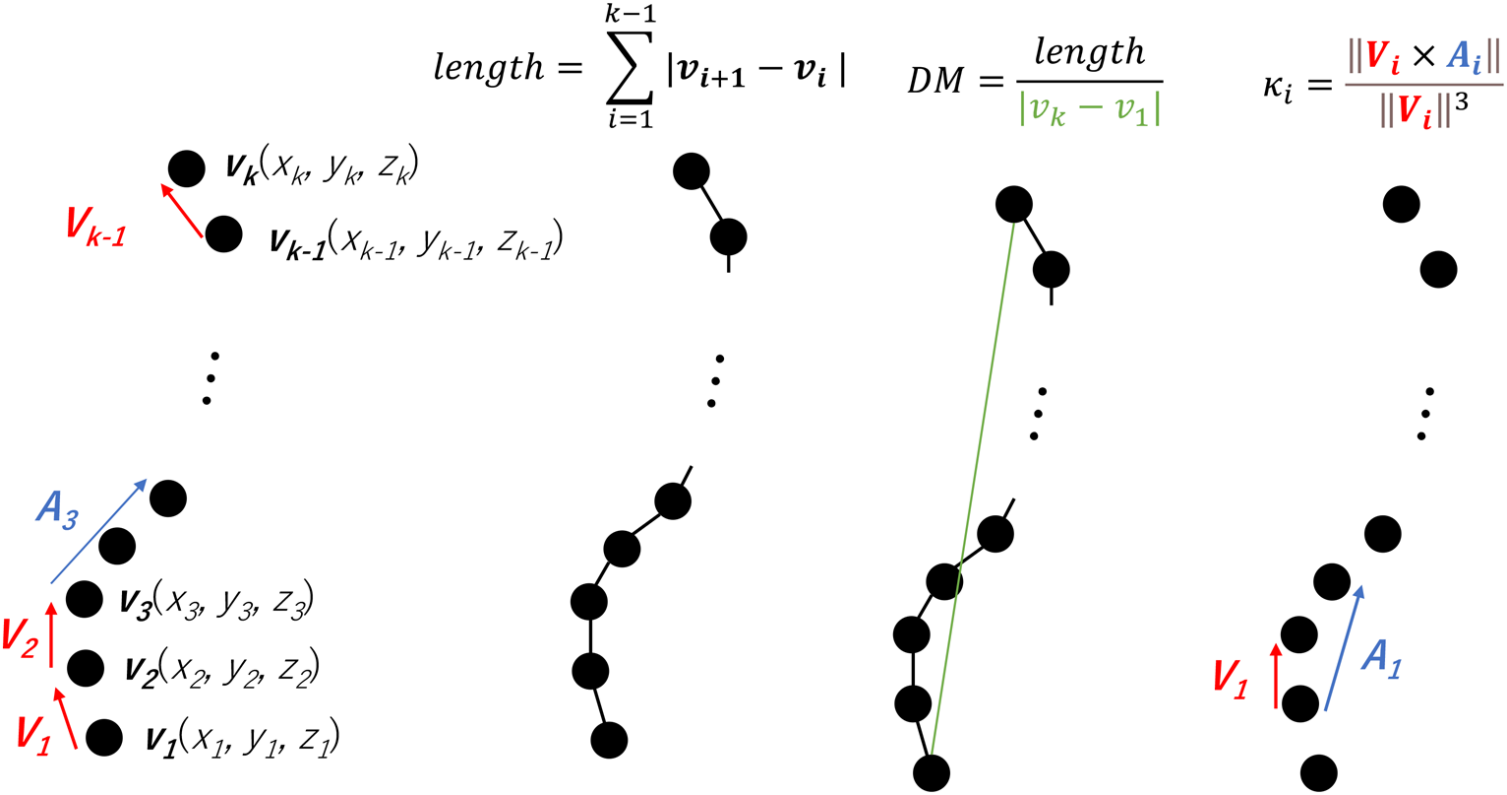
An explanation figure for each vector and geometric feature in this study.

The geometric features of arteries were quantified based on the Frenet–Serret formula [20] and the Bullitt et al. definition [21] as summarized in Figure 2. We define the vector ***v*** by extracting the three‐dimensional coordinates from the vessel centerline, ***p***, while excluding the radius components. Each component *x*_*i*_, *y*_*i*_, *z*_*i*_ of *v* is derived from *p* as follows: *x*_*i*_ is taken from the first *k* entries, *y*_*i*_ from the next *k* entries, and *z*_*i*_ from the next *k* entries specifically $v_{i}=\left(x_{i},y_{i},z_{i}\right)$ for i=1,2,…,k, thus forming a vector of spatial coordinates without consideration of their respective radii.

The velocity vector ***V*** at point ***v***_***i***_ (*x*_*i*_, *y*_*i*_, *z*_*i*_) can be approximated by the vector between the points ***v***_*i*_ and ***v***_*i*+1_.

(7) $$\begin{array}{c} V_{i}=v_{i+1}-v_{i} \end{array}.$$

Acceleration vectors ***A*** are approximated from ***V***_***i***_ as:

(8) $$\begin{array}{c} A_{i}=V_{i+1}-V_{i}, \end{array}$$

Maximum local curvature: Local curvature κ_*i*_ is defined as follows.

(9) $$\begin{array}{c} \kappa_{i}=\frac{\left\|V_{i}\times A_{i}\right\|}{{\left\|V_{i}\right\|}^{3}} \end{array}.$$

Hence, maximum local curvature is defined by the maximum value of κ_*i*_ as expressed by:

(10) $$\begin{array}{c} \text{maximum local curvature}=\max\left(\kappa_{i}\right). \end{array}$$

Length: The vector length [21] is defined as:

(11) $$\begin{array}{c} \text{length}=\sum_{i=1}^{k-1}\left|v_{i+1}-v_{i}\right|. \end{array}$$

Distance metric: The distance metric (DM) is the geometric feature that describes a ratio between the actual course length of a meandering curve and the linear distance between endpoints [21]. The distance metric produces a dimensionless number [21] and is defined as (12).

(12) $$\begin{array}{c} \mathrm{DM}=\frac{\sum_{i=1}^{k-1}\left|v_{i+1}-v_{i}\;\right|}{|v_{k}-v_{1}|} \end{array}.$$

#### 2.3.2 Evaluate Goodness of Fit According to the Number of Landmark Points

To examine the character of the VPs generated by MVND, we plotted the distribution of geometric features (length, DM, and maximum local curvature) as histograms. KL divergence was employed to quantify the fitness of our distribution of VP's geometric features against the distribution of the RP's geometric features [15], [22]. Since KL divergence quantifies the difference of two distributions on each bin, KL divergence responds to local shape difference. To address the imbalance of sample size in RPs and VPs, which complicates direct comparison of distribution shapes, these histograms were converted to relative frequency histograms. The bin size was fixed across all features, with the number of bins set at 30.

For two features, x_r_ and x_v_, from the RPs and VPs, respectively, with histograms, $f_{x_{r}}$ and $f_{x_{v}}$ defined on the same probability space, the KL divergence [16] quantifies the divergence between the two distributions, in an asymmetric manner, as in:

(13) $$\begin{array}{c} \mathrm{KL}\left(f_{x_{r}}\|f_{x_{v}}\right)=\sum_{i=1}^{I}f_{x_{v}}\left(k_{i}\right)\log\left(\frac{f_{x_{v}}\left(k_{i}\right)}{f_{x_{r}}\left(k_{i}\right)}\right) \end{array},$$

where KL values close to 0 denote that the two histograms $f_{x_{r}}$ and $f_{x_{v}}$ are almost identical in terms of highly reduced divergence or highly increased convergence, and *I* is equal to the number of bins. This KL divergence represents the “distance” from the geometric feature distribution of the real population (ground truth) to that of the Vpop.

In addition to the quantitative comparison using KL divergence, qualitative evaluation was performed to confirm the plausibility of anatomical characteristics and their diversity of the VPs by visual inspection.

#### 2.3.3 Change the Number of Landmark Points

In this procedure, we examined the change of KL divergence value from the number of landmark points in order to further examine the effect of the number of landmark points to be extracted. The number of landmark points was set in the range 3 to 60 for ICA and in the range 3 to30 for BA. At each number of landmark points, 100 VPs generation were performed 30 times independently. Afterwards, KL divergences were calculated for each trial. The mean and standard deviation of KL divergence were calculated for each number of landmark points.

## 3 Results

### 3.1 Centerline Visualization

**FIGURE 3:**
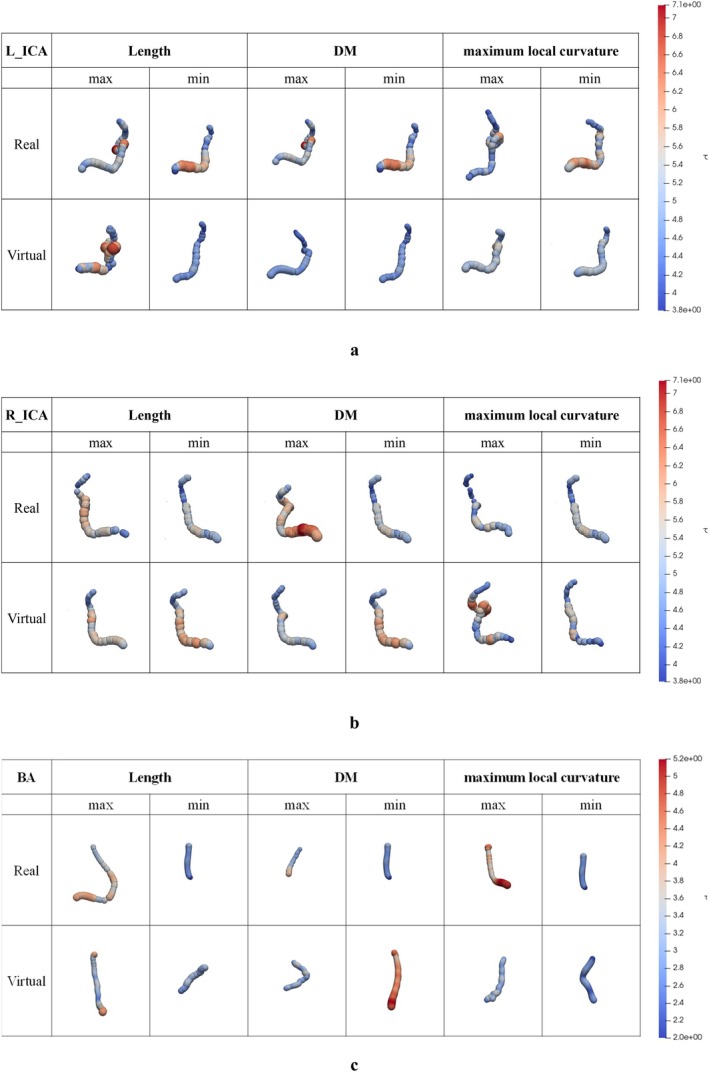
The visualization result of PRs and VPs by posterior–anterior view.

The internal carotid artery (ICA) typically appears as a smooth, tubular vessel with an ascending cervical segment, followed by a curved petrous segment as it enters the skull base [23]. It then passes through the cavernous sinus, often taking an S‐shaped path, and continues intracranially as a straight supraclinoid segment. Figure 3 presents the visualization results for the L_ICA, R_ICA, and BA. For each region, the arteries with the minimum and maximum values of length, DM, and maximum local curvature are shown. In addition to a quantitative evaluation of the shape, a comparison of the appearance was made in order to intuitively assess whether the appearance reproduced the features specific to the actual vessel shape. Both VPs and RPs are displayed, allowing for direct comparison. The visualizations shown here are based on trial 1, with 30 landmark points for the BA and 60 landmark points for the L_ICA and R_ICA, respectively.

**FIGURE 4:**
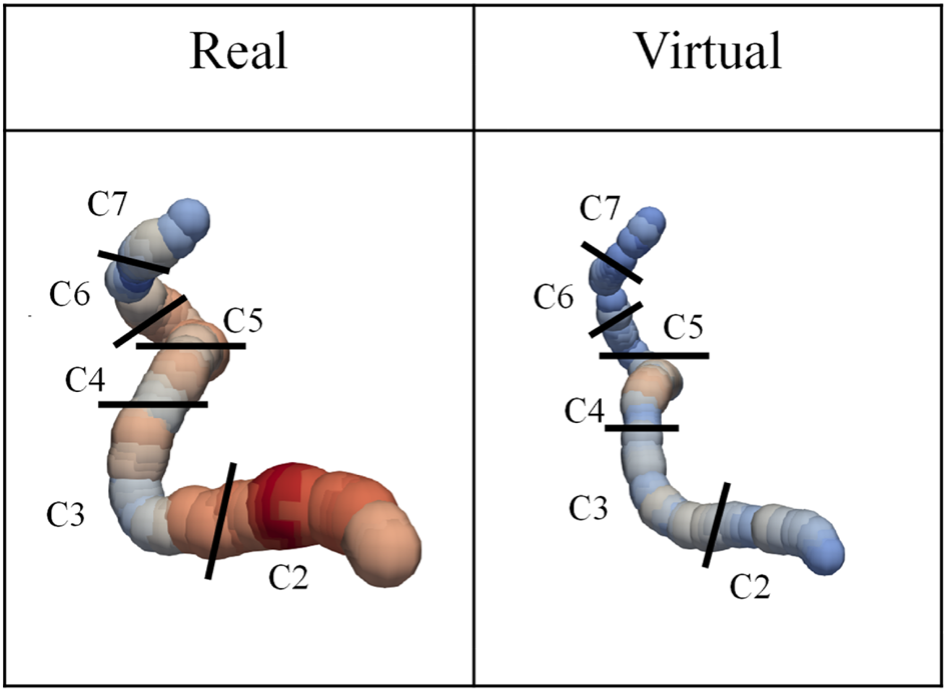
The real and virtual R_ICA visualization, which has the maximum value of DM among each population.

In ICAs, the term ‘siphon’ refers to the characteristic S‐shaped curve of the ICA, typically found in C4–C5 segments shown in Figure 4. This curvature plays a role in dampening pulsatile blood flow and is a distinctive anatomical feature of the ICA. In some patients in both RPs and VPs, particularly those with shorter ICA lengths or lower curvature, this siphon shape occasionally showed absence. The RP's shape of BA with the value of maximum curvature tends to be curved, while the virtual BA with the minimum value of curvature tends to be straight. The VP's BA reproduces these tendencies. Therefore, VPs reproduce similar characteristics to RPs in terms of centerline course.

### 3.2 Length

The average and standard deviation of length are summarized in Table 2. The average length of ICA in RPs and VPs is found to be approximately the same; the average length of L_ICA is 7.92 ± 0.718 cm (RPs) and 7.82 ± 0.939 cm (VPs). For a reference for the average length of ICA, Baz et al. reported the length of ICA (C2–C7), which is almost the same region as the present study, was 6.94 cm (right side) and 6.933 cm (left side) [24]. Note that the definition of segments is not defined using some algorithm/equation. Hence the length might vary among studies. (Centerline data in the present study was constructed from head MRA and the exact definition of starting points of C2 segments might differ from their study.)

**TABLE 2:** The average length and its S.D. among literature and real and virtual at each part.

	L_ICA [cm]	R_ICA [cm]	BA [cm]
Real	7.92 ± 0.718	7.99 ± 0.753	3.13 ± 0.832
Virtual	7.82 ± 0.939	7.99 ± 0.762	3.16 ± 0.545
Baz et al. [24]	6.93 ± 0.842	6.94 ± 0.895	
Kalaiyarasi et al. [25]			2.792 ± 0.32
Wankhede et al. [26]			2.99 ± 0.29
Nishikata et al. [27]			2.46 ± 0.436 (male) 2.33 ± 0.401 (female)

For the average length of both the RP's and VP's BA, their values were found to be close to each other; the average length of BA is 3.19 ± 0.832 cm (RPs) and 3.16 ± 0.545 cm (VPs). Also for general reference for the average length of BA, three studies were considered in Table 2 [24, 25, 26, 27]. The definition of BA was based on bifurcation from the vertebral artery to the posterior cerebral arteries in both the present study and previous studies. The average length of the RP's and VP's BA reported here was generally higher than the average length of BA reported in the earlier studies.

### 3.3 Inner Diameter

The average diameter of both RPs and VPs was found to be approximately the same in both L_ICA and R_ICA as shown in Table 3; the L_ICA are 5.42 ± 1.02 mm (RPs) and 5.23 ± 1.04 mm (VPs) while the R_ICA are 5.24 ± 0.624 mm (RPs) and 5.27 ± 0.780 mm (VPs). Overall, compared with the RP's and VP's ICA diameter average and standard deviation, their diameters seem to be in good general agreement with the literature range [28, 29, 30].

**TABLE 3:** The diameter averages and its S.D. between and real and virtual at each part.

	L_ICA [mm]	R_ICA [mm]	BA [mm]
Real	5.42 ± 1.02	5.24 ± 0.624	3.33 ± 0.675
Virtual	5.23 ± 1.04	5.27 ± 0.780	3.41 ± 0.653
Choudhry et al. [28]	4.7 ± 0.70	4.8 ± 0.70	
Ojaare et al. [29]	4.61 ± 0.63	4.63 ± 0.63	
Krejza et al. [30]	5.11 ± 0.87 (male) 4.66 ± 0.78 (female)		
Smoker et al. [31]			3.17 ± 0.726
Lam et al. [32]			3.7 ± 0.9
Vijayakumar et al. [33]			3.7 (ranged from 2 to 5 mm)

Also, the average diameters of the BA in the RPs and VPs were found to be approximately the same; the average diameter of BA is 3.33 ± 0.675 mm (RPs) and 3.41 ± 0.653 mm (VPs). For general reference for the average BA diameter, three studies were considered [31, 32, 33]. Compared with the real and virtual BA diameter average and standard deviation, their diameters seem to be in good general agreement with the literature range.

In the RP's ICA, the inner radius of the artery exhibits significant variability, characterized by an irregular or undulating contour along the course of the ICA, with notable changes in calibre (or a “beaded” appearance) observed throughout its course as shown in Figure 2a,b. The virtual ICA reproduces that characteristic. These characteristics are both consistent with the large standard deviation shown in Table 3. Also, in the RP's BA, the inner radius of the artery is almost the same as this ICA characteristic observed throughout its course as shown in Figure 2. The VP's BA reproduced that characteristic.

### 3.4 The Distributions of Geometric Features

**FIGURE 5:**
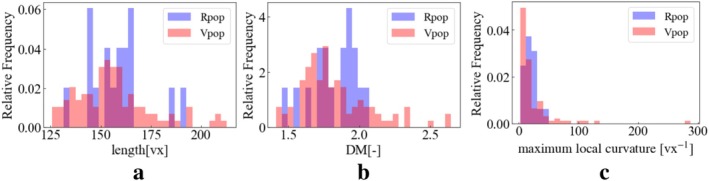
Real and virtual L_ICA's geometric features distribution at 60 landmark points on trial 1. (a) Length (b) DM (c) maximum local curvature.

**FIGURE 6:**
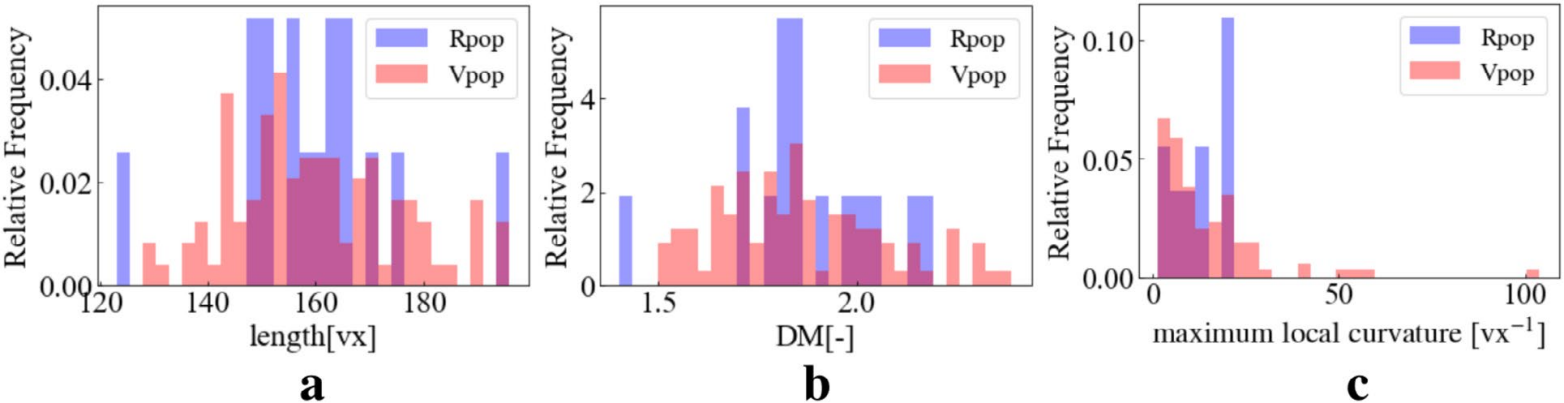
Real and virtual R_ICA's geometric feature distribution at 60 landmark points on trial 1. (a) Length (b) DM (c) maximum local curvature.

**FIGURE 7:**
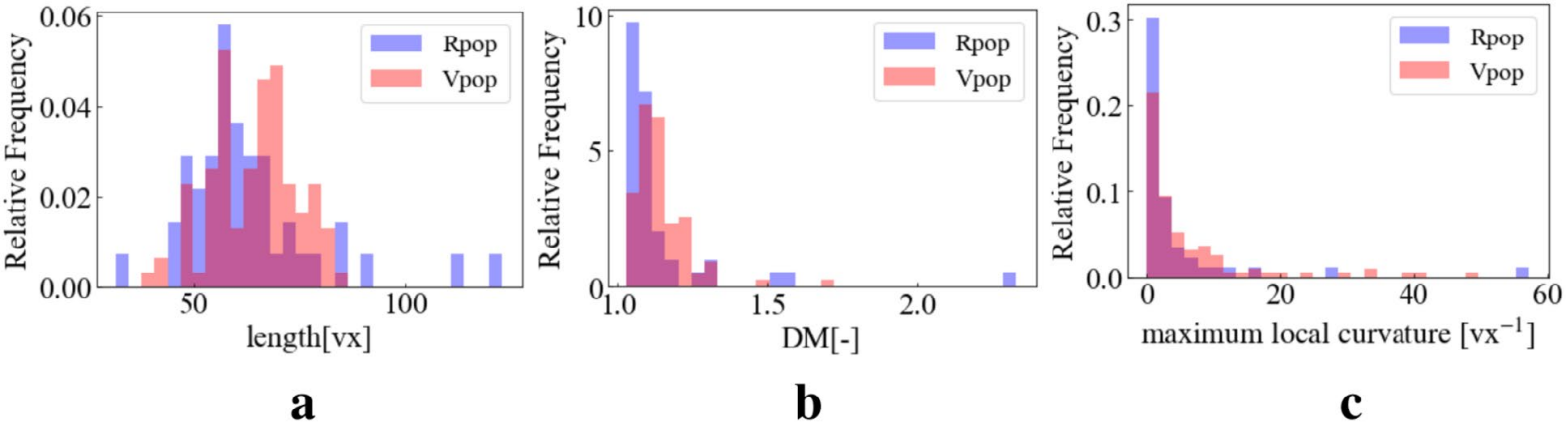
Real and virtual BA's geometric features' distribution at 30 landmark points on trial 1 (a) Length (b) DM (c) maximum local curvature.

Figures 5, 6, 7 display the overlay of distributions for each geometric feature in RPs and VPs. We visualized the distributions specifically focusing on cases with 30 landmark points for the BA and 60 landmark points for the ICA. Note that this is 1st trial data of 30 times trial in 100 generation.

Both RPs and VPs exhibit a peaked distribution. The peak of the VPs distribution tends to shift to a lower value (left side) compared to the RPs distribution in DM and maximum local curvature. The length distribution in VPs has two peaks. The mid‐point between the two peaks in VPs is the same as the RPs' peak point.

In the RP's geometric features, the distributions appear quite sparse in some regions. This sparsity can primarily be attributed to a smaller sample size for the real patients compared to that of the VP's ones, which limits the representation of the full range of geometric variations in the RPs. Conversely, the VP's distributions exhibit a continuous distribution compared to their real counterparts. This increased continuous distribution is a consequence of the larger sample size used in generating the virtual models. The substantial sample size allows for a more comprehensive and continuous representation of the geometric features across the spectrum.

### 3.5 Kullback–Leibler Divergence

**FIGURE 8:**
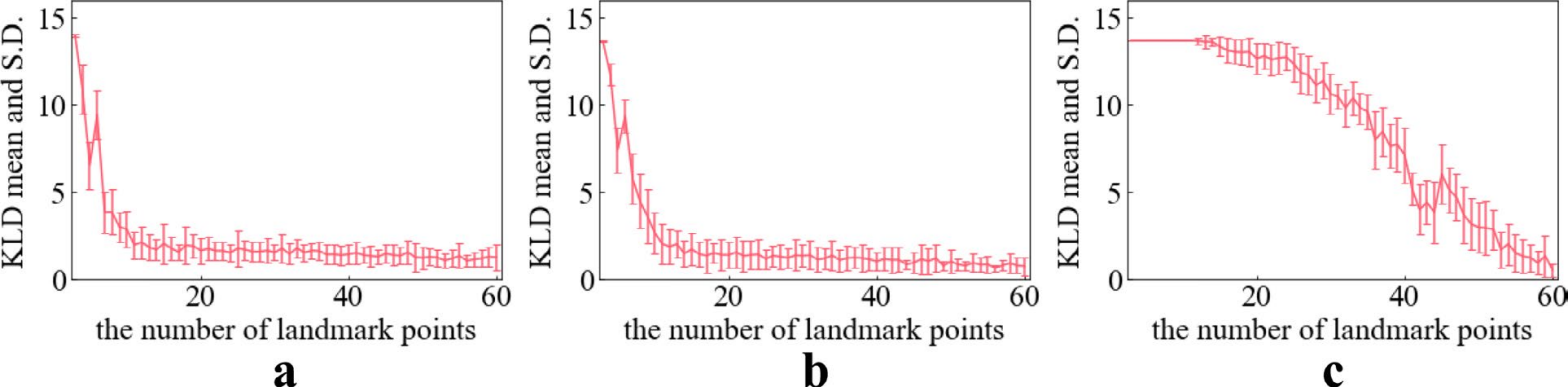
KL divergence mean and S.D. tendency on L_ICA. (a) Length (b) DM (c) maximum local curvature.

**FIGURE 9:**
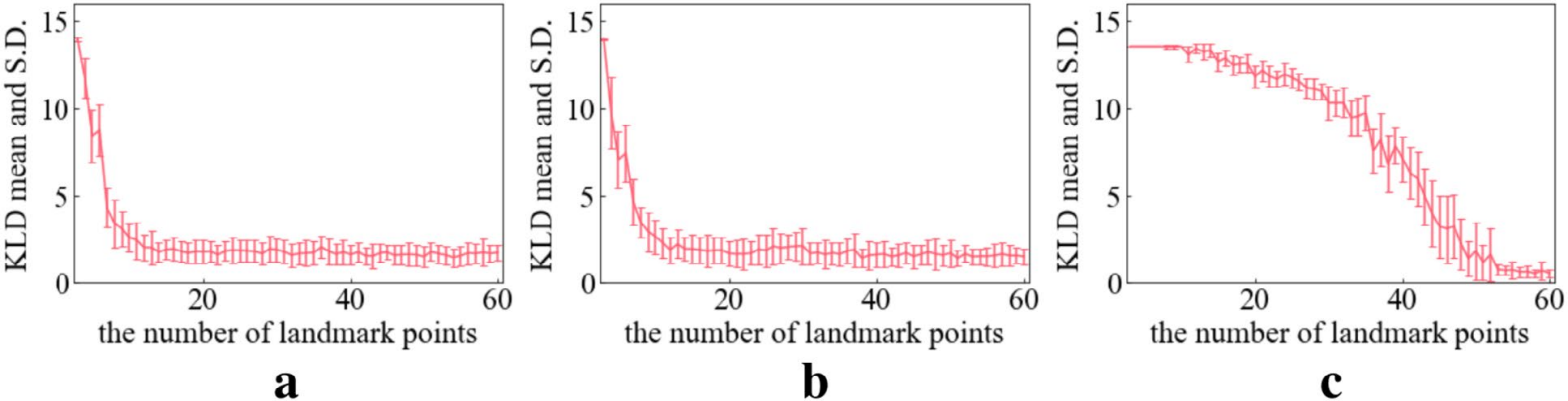
KL divergence mean and S.D. tendency on R_ICA. (a) Length (b) DM (c) maximum local curvature.

**FIGURE 10:**
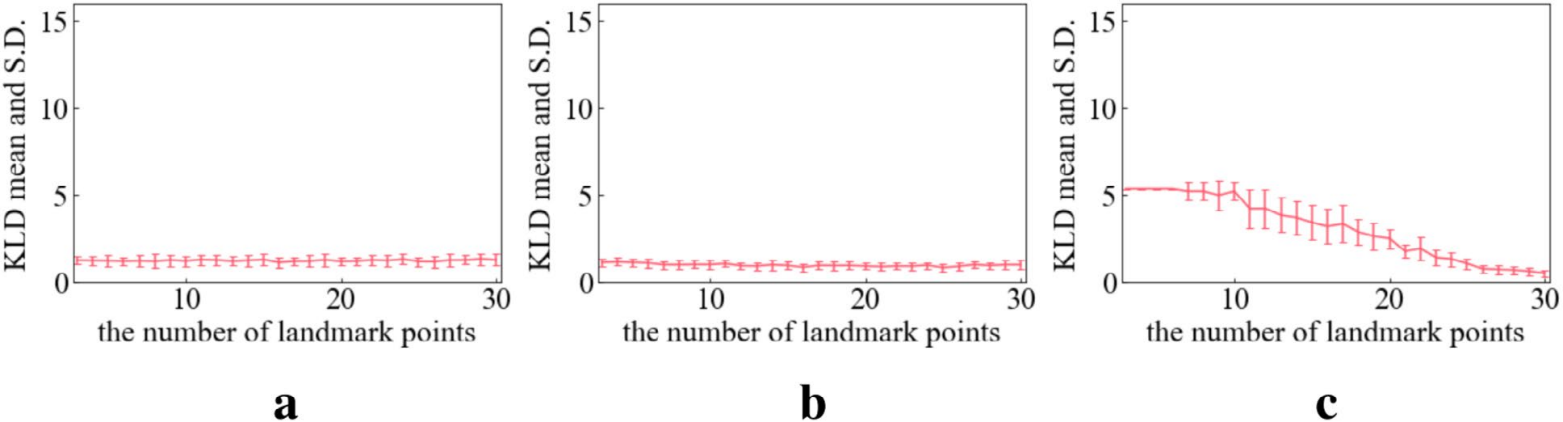
KL divergence mean and S.D. tendency on BA. (a) Length (b) DM (c) maximum local curvature.

Figures 8, 9, 10 show the KL divergence for the distribution of the three geometric features. Lower values of KL divergence mean better agreement between the RP and VP distributions of their geometric features. These figures show the relationship between the KL divergence values and the number of landmark points.

In the case of the length of the L_ICA and R_ICA, a notable decrease in the KL divergence is seen when the number of extraction points is around 10 (Figures 8 and 9a). Following this drop, the KL divergence mean values stabilize between 0 and 2.5, suggesting a good quality fit between the virtual and real distributions.

In the case of DM of L_ICA and R_ICA, a notable decrease in the KL divergence is seen when the number of extraction points is around 10 (Figures 8 and 9b). Following this sharp decrease, the KL divergence mean values stabilize between 0 and 2.5, suggesting a good fit between the VP's and RP's distributions.

For the maximum local curvature for the L_ICA and R_ICA, the KL divergence mean value remains consistently high until about 10 extraction points (Figures 8 and 9c) and then exhibits a gradual decrease. As the number of landmark points increased (*N* = 3–60), the KL divergence tends to decrease, indicating a progressively improving fit between the two distributions.

The KL divergence in BA shows a different tendency than that of ICAs. The KL divergence values in length and DM remain almost constant irrespective of the increase in the number of extraction points (Figure 10a,b). This suggests that the increase in landmark points does not affect the fitness of distributions significantly. For the maximum local curvature of the BA (Figure 10c), with more than around 10 landmark points, the mean value of the KL divergence gradually decreases with each additional landmark point.

## 4 Discussion

Our goal was to develop a simple MVND method for generating a population of distributed virtual arteries with multiple geometric features. The results of the fitting of the distribution of geometric features indicate that if enough landmark points are used, the MVND method can reproduce the complexity of an artery course and the diversity of the geometric features. Considering the correlations among all positional and inner radius variables, the MVND method may automatically incorporate the continuity of the vascular centerline and the anatomical characteristics of cerebrovascular geometries. This suggests that the MVND method with enough landmark points can generate VPs more simply than the conventional studies.

### 4.1 Length and Inner Diameter

Regarding the length of the ICA in Table 2, the RP and VP average length values reveal excellent agreement between them. This suggests that the MVND method has an ability to reproduce the geometric feature of ICA's complexity and diversity. Considering the average length reported in earlier studies, the lengths of the RPs and VPs in this study also show good agreement. In a while, the length of RP and VP is slightly higher than those reported by previous studies [24, 25, 26, 27]. This is likely because the degree of the B‐spline, and the strength of smoothing may measure the centerlines longer compared to earlier studies. Following a similar line of argument, focusing solely on the RP and VP average length of the BA, the values and their variability appear to be very similar. The average diameter for both BA and ICA showed excellent agreement among RPs, VPs, and literature values [28, 29, 30, 31, 32, 33] as shown in Table 3. These results suggest that the MVND method has the potential to reproduce VPs closely resembling the values reported in the literature, provided that an appropriate RP is used as the basis.

Both RPs and VPs share a similar characteristic, with diameters that can vary significantly in calibre, appearing “beaded” in shape. This might be due to image resolution; when calculating arterial radius from MRA in this study, the spatial resolution was about 0.5 mm per voxel. Compared to the diameter of cerebral arteries (3–5 mm), this 0.5 mm resolution is likely to cause frequent changes in diameter along the course of RPs, and the MVND method will reproduce this characteristic in VPs. Higher resolution or some form of smoothing will be needed to decrease the beaded characteristics, and to enhance the plausibility of VPs.

### 4.2 Quantifying the Difference of Geometric Feature's Distributions

Our objective was to develop a generation method for VPs, which can have a similar distribution to RPs, using a MVND. To evaluate this method, we compared it to the distribution of RPs using the KL divergence metric. There are other metrics such as the KS test [22] and Wasserstein distance [34], and it is possible to estimate which part of the histogram each formula is sensitive to. We applied KL divergence, the KS test and the Wasserstein distance in our past study [35]; the KL divergence excels in detecting differences in distribution shapes in each bin, the KS test offers a robust non‐parametric comparison that detects the difference of maximum distance, and the Wasserstein distance provides an intuitive measure of the distributional distance. Hence, KL divergence responds to the local shape difference. In the previous study [35], KL divergence started decreasing around ten landmark points, while the KS test started the response around fifty landmark points. As a result, KL divergence with various numbers of landmark points enables detection of the transient evolution of distribution fit.

From Figures 8 and 10, importantly, the KL divergence demonstrated that increasing the number of landmark points improved the fit of RP's and VP's geometric features distribution. This suggests that an enough number of landmark points in the MVND method can contribute to reproducing the diversity and complexity of RPs such as length, DM, and maximum local curvature; length and DM represent global features of vascular shape, while maximum local curvature reflects local features between adjacent points of the vascular centerline (interpolation points). The fit of the global feature distributions such as length and DM responds broadly to changes in the number of landmark points, whereas that of the local feature, maximum local curvature, reacts more sensitively to the changes of the number of landmark points. This indicates that the fit of global features distribution between RPs and VPs is brought with fewer number of landmark points (around 10 landmark points) and that of local features distributions is brought with more landmark points (around more than 60). Therefore, approximately 10 landmark points are necessary to accurately replicate the global complexity and the diversity of the ICA represented by length and DM. An et al. used about 6 inflection points among these segments [36]. Consequently, to replicate these inflection points, it is necessary to set at least 8 control points when using a second‐order B‐spline, as shown by [19, 37]. This means that for an ICA with 6 inflection points, more than 11 landmark points (knots) are required as a minimum. Interestingly, the values of length and KL divergence in L_ and R_ICA start to provide a better estimate when the number of extraction points exceeds 11 landmark points.

A similar discussion applies to the BA. Due to its inherently simple curved shape, the number of landmark points does not significantly affect the reproducibility of the distributions of global features, such as length and DM. In contrast, for reproducing the distribution of local features, such as maximum local curvature, the fit begins to stabilize only when more than 10 landmark points are used.

In this study, landmark points were set at equal intervals, and we set landmark points according to segments from C2 to C7. The ICA is commonly divided into several segments: from C1 to C7 segment. Note that these segments do not have equal length among individuals. Certain segments may exhibit longer or shorter lengths depending on individual anatomical variations. Consequently, the distribution of landmark points across segments may differ among individuals. Segment‐based landmark placement will facilitate more precise reproduction of anatomical shapes, necessitating the incorporation of segment annotation techniques.

Regarding the KL divergence value observed in this analysis, while some previous studies have evaluated distributions using KL divergence for parameters such as age and gender [22], to our best knowledge, no studies have yet applied this metric to assess 3D shape diversity. Additionally, the value of KL divergence is dependent on sample size, making it difficult to directly compare the results of previous studies with those of the present study.

In summary, if the objective is to reproduce only the distributions of global geometric features, such as length and DM, for the ICA and BA, approximately 10 landmark points may suffice. However, for both the ICA and BA, if the goal is to accurately capture the distributions of both global and local features, it is recommended to set as many landmark points as possible.

### 4.3 Contribution of MVND to Generate the Virtual Arteries

As the MVND method incorporates the mean and covariance matrices, it can encapsulate the correlation of multiple elements and can model the distribution patterns of variables (*x, y, z, r*) when enough number of landmark points is considered. Hence, it is likely effective to capture the complexity and diversity of RPs, and then VP generation with MVND can generate shape variations by using a—when computational simplicity and interpretability are desired, and when the variability is suitably captured by Gaussian assumptions. PCA‐SSM is advantageous for uncovering major population trends from observed examples but is less flexible outside the observed data space. GPMMs provide maximal adaptability and anatomical fidelity at the cost of significantly increased complexity and computational burden.

In the present study, the variables (*x, y, z, r*) correlate with each other based on the following two anatomical reasons. Firstly, artery courses have a regular pattern in brain space; for example, the ICA fits to the carotid canal, and the BA fits to the pons. ICAs have syphon shapes and always move through similar coordinates, while BAs often have shapes that curve convexly downward along the pons. Secondly, blood vessels must exhibit continuity. The arteries course continuously; therefore neighbors should be located near each other even between different patients. In other words, neighbors have similar coordinate values. Due to the anatomical constraints above, the distribution patterns of variables (*x, y, z, r*) indicate that each section of the artery maintains a certain spatial relationship (proximity) with other sections, and this relationship can be represented systematically.

This correlation, observed at the individual level, can also be extended to populations. The regularity in arterial courses and the continuity of blood vessels are consistent across individuals, indicating that these anatomical patterns are preserved on a broader scale. If a specific arterial segment is consistently located in close proximity across multiple patients, and its relative spatial relationship remains consistent. Therefore, random sampling from the MVND can generate a VPs that follows the distribution of the RPs.

### 4.4 Limitation

In this study, the (*x, y, z, r*) parameters were modelled assuming a MVND; however, actual human anatomical shapes may not always conform to MVND. For instance, the x‐coordinate values of the BA may not adhere to a single normal distribution. The BA tends to curve to one side rather than remaining straight in the middle, as noted by [38, 39]. The location of the BA varies considerably because it is surrounded by soft tissues such as the pons, which contribute to greater variability in its positioning. In future work, it will be necessary to verify the assumption of multivariate normality using a larger sample size and conducting tests [40] such as Mardia's test [41], Henze and Zirkler test [42], Royston's test [43], or Energy test [44]. Therefore, a larger patient dataset will be required to test for normality. The sample size of patients should exceed the total number of variables, which is calculated as the number of landmarks multiplied by four (*x*, *y*, *z*, *r*):to validate the ICA with 60 landmark points, 240 patients would have been required. For instance, the BraVa dataset, an open‐source MR‐based centerline dataset, consists of 62 healthy subjects. By combining other open datasets, this requirement for the normality test can be examined, but it is still challenging to maintain the quality and format of datasets from various sources. If larger datasets become available in the future, validation for this assumption can be achieved.

Regarding the evaluation of distribution fitness, the BA exhibits low KL divergence across the entire range of landmark points for both length and DM. Since the BA typically consists of one or two curves, making its geometric characteristics relatively simple, geometric features in RPs and VPs will become more similar. However, for example, the asymmetry of the BA described above cannot be assessed with current geometric features. Hence, other geometric features will be required to more precisely evaluate the complexity and diversity of arteries.

Previous studies have established complex exclusion criteria to exclude physiologically improbable VPs when generating VPs. For example, Niederer et al. generated VPs by random parameter sampling [45]. They compared the distribution of VPs with the RP dataset using principal component analysis (PCA) and discarded models that deviated significantly from the actual physiological parameter space, thereby eliminating unrealistic VPs. Thamsen et al. generated VPs for vascular shapes using PCA‐SSM and eliminated those whose geometric features deviated from the range reported in clinical reports [10]. In the present study, the resampled variable (such as diameter) could be negative in MVNDs theoretically as well as in the earlier proposed methods, although this was not found to be an issue in the present study. Nevertheless, in future studies to generate the VPs with specific character, it may be necessary to explore methods for ensuring that sampled variables fall within the intended ranges.

In this study, landmark points were set as equal space sampling. Kjeldsberg et al. conducted a sensitivity analysis of the sampling length of landmark points, and they concluded that dense landmark points lead to higher reproducibility of artery complex shapes [46], which shows a similar trend to the present study. Meanwhile, the other sampling methods might lead to different results, such as anatomy‐based landmarking. Tan et al. applied a deep‐learning‐based algorithm which automatically detects bifurcation points on brain networks [47]. To apply anatomy‐based landmarking to represent the complex shape of curved arteries, automatic segmentation of curves [46] based on curvature may be required.

The current study implemented B‐spline interpolation to fill the gaps between landmark points. The number of interpolation points was set to match the average number of RPs, and the VPs were set to be the same as the RPs. A second‐order B‐spline interpolation was applied. Minor variations in these settings could lead to changes in the reconstructed vascular shape. As mentioned in the results, applying smoothing through interpolation can shorten the lengths of arteries in both RPs and VPs. Consequently, the lengths of RPs and VPs in the present study become comparable to those reported in reference articles (Table 2). In any case, MVND generates VPs that reflect the characteristics of the RPs.

## 5 Conclusion

The MVND method was proposed to develop a set of virtual cerebral vascular data that reproduces the geometric feature distribution of vascular course. In addition, MVND is a mathematically simple modeling approach. Qualitatively, VPs and RPs exhibit very similar characteristics, suggesting that the MVND, by considering the correlations among all positional and inner radius variables, may automatically incorporate the continuity of the vascular centerline and the anatomical characteristics of cerebrovascular geometries without any parameter tuning. Once the number of landmark points exceeded 10, the global geometric feature distribution of the ICA began to exhibit the fit in terms of KL divergence, which suggests that the number of landmark points must be sufficient to capture the complexity of the ICA's shape. In contrast, for the BA, which exhibits a relatively simple trajectory, the KL divergence was less influenced by the number of landmark points. These findings suggest that if the necessary number of landmark points is used to capture the complexity of vascular shape, it may be possible to generate VPs more simply than with conventional methods.

## Conflicts of Interest

The authors declare no conflicts of interest.

## Supporting information


**Data S1:** cnm70117‐sup‐0001‐Supinfo.docx.

## Data Availability

Data sharing is not applicable to this article as no datasets were generated or analysed during the current study.
