# Supplementary material for: Multivariate Normal Distribution Method for a Virtual Cerebral Arterial Population
Source: Int J Numer Method Biomed Eng. 2025 Nov 15;41(11):e70117. doi: 10.1002/cnm.70117 (PMC12619570; doi:10.1002/cnm.70117)
Supplement: Supplementary file 1 — Data S1: cnm70117‐sup‐0001‐Supinfo.docx. [file CNM-41-e70117-s001.docx]

**Supplementary materials**

**Construction of centerline data from MRA images**

The database comprised 46 hypertensive patients aged 31-76 years (56.29 ± 10.63 years, 27 men and 19 women) with primary aldosteronism (PA). The images were obtained by using the time-of-flight method on magnetic resonance (MR) scanners (GE Medical Systems, SIEMENS, Philips Medical Systems, or Toshiba) and exported in DICOM format. The resolution of the MR scanners ranged from 0.28 mm to 0.41 mm. Slice thickness ranged from 0.40 mm to 0.65 mm.

Then, linear registrations of shift, rotation, scale, and shear were performed to minimize the differences between the object image and the referenced image of MNI152 by using the FMRIB software Library v6.0 (FSL, Oxford Univ.). The image was 364 × 436 × 364 [vx], with an isotropic voxel size of 0.5mm.

After linear registration, an arterial volume was segmented by using a thresholding method. Segmentation using the threshold method was performed on the registered patient images. The intensity threshold was set at 99.5%, which is the intensity at which most main arteries can be identified. Segments with more than 1000 voxels connected from the extracted high-intensity regions were then considered as arteries. Skeletoning was performed on the segmentation, and centerlines for all arteries were extracted using python scikit-image library.

Then, centerlines were determined for each arterial segment on the basis of the bifurcation location. The diameter was defined as the diameter of an inscribed sphere at each centerline point.
